# Development of A novel ferroptosis-related prognostic signature with multiple significance in paediatric neuroblastoma

**DOI:** 10.3389/fped.2023.1067187

**Published:** 2023-02-22

**Authors:** Xin Wang, Jun Yang, Hongqiang Bian, Hu Yang

**Affiliations:** Department of General Surgery, Wuhan Children’ Hospital, Tongji Medical College, Huazhong University of Science and Technology, Wuhan, China

**Keywords:** ferroptosis, neuroblastoma, prognosis, immunotherapy, tumor microenvironment

## Abstract

**Background:**

Ferroptosis is an iron-dependent regulated cell death pathway that plays an essential role in the occurrence and development of tumours. Nonetheless, little is known about the impact of ferroptosis-related genes (FRGs) on neuroblastoma.

**Methods:**

Transcriptional profiles and clinicopathological data of neuroblastoma were downloaded from the TARGET and GEO datasets. These were used as the training set and the validation set, respectively. Non-negative matrix factorisation was employed to divide patients with neuroblastoma into distinct ferroptosis clusters. The Cox regression model with LASSO was performed based on the FRGs to construct a multigene signature, which was subsequently evaluated in the testing set. Finally, we analysed the differences in the tumour immune microenvironment (TIME) and immunotherapeutic response among the different risk groups.

**Results:**

The two distinct ferroptosis subtypes were determined and correlated with different clinical outcomes and tumour-infiltrating immune cells (TIICs). A risk model was developed to explore the risk scores of the individual patients. Patients in the low-risk group survived significantly longer than those in the high-risk group and showed a good predictive performance in the testing set. The risk score was significantly linked to clinicopathological traits, and it was confirmed as an independent prognostic indicator for assessing the overall survival. We also found that patients with low-risk scores had a higher infiltration of TIICs and a better immunotherapeutic response.

**Conclusions:**

This study showed the potential role of FRGs in contributing to the clinical features, prognosis, TIME, and immunotherapy of neuroblastoma cases. Our findings offer a valuable basis for future research in targeting ferroptosis and its TIME and provide novel measures for the prevention and treatment of neuroblastoma.

## Introduction

Neuroblastoma (NB) is a cancer that affects neural crest-derived cells and can occur anywhere in the sympathetic nervous system (1). It is one of the most common extracranial solid tumours in children, accounting for 15% of all childhood cancer-related deaths (2). Most patients are under 5 years of age at diagnosis, with a median age of 17–18 months (2, 3). Patients were classified into risk groups based on molecular factors and disease presentation, including age, tumour histology, local vs. metastatic disease, and genomic alterations (4). Low-risk patients have a good prognosis with a 5-year survival rate of >90%. However, most neuroblastomas are diagnosed as high-risk, and most of these patients have metastases at the time of diagnosis and have a very poor prognosis, with 5-year survival rates below 50% (2, 5). Patients with low-risk NB are usually treated surgically or may experience spontaneous tumour regression. Patients in the moderate-risk group received milder chemotherapy or underwent resection of the remaining tumour mass. The standard treatment for high-risk NB consists of induction, consolidation, and maintenance of the three treatment blocks. In addition, the responses to current standard therapies are highly heterogeneous, ranging from complete regression to multidrug resistance and severe toxicity (6). NB is a complex disease that exhibits biological, clinical, morphological, and genetic heterogeneity, making the development of a successful generic treatment very difficult (6). Hence, it is of great significance to reveal the heterogeneity of NB and screen novel prognostic indicators and therapeutic schedules to improve the survival rate of patients with NB.

Ferroptosis is a new cell death pattern with distinct genetic and biochemical features, including iron dependence, which differentiates it from apoptosis and autophagy (7–10). The deactivation of glutathione peroxidase-4 is the primary mechanism of ferroptosis, which can cause an increase in lipid peroxidation and glutathione consumption (9, 10). Recently, researchers have found that ferroptosis can inhibit tumour independent of other classical tumour inhibition mechanisms (9). In recent years, the induction of ferroptosis has become a promising treatment alternative to cancer cell apoptosis, particularly in tumours that are not responsive to popular treatments (11, 12). However, little is known about the regulation of ferroptosis during cancer progression and the specific ferroptosis-related genes (FRGs) that play an essential role in NB. Therefore, further research on the potential role of FRGs in patients with NB will help identify new targets for NB treatment and novel predictive prognostic marker genes.

In this study, we integrated Therapeutically Applicable Research to Generate Effective Treatments (TARGET) and GEO databases to assess the links between ferroptosis patterns and characteristics of the tumor microenvironment (TME). We provided substantial evidence that neuroblastoma can be categorised into two subtypes with distinct survival and immune patterns using unsupervised clustering. Subsequently, a ferroptosis risk model was established to assess the risk scores of the individual patients. Furthermore, we analysed the potential relationships between ferroptosis and clinical outcomes, TME, immune checkpoints, and immunotherapeutic responses.

## Materials and methods

### Acquisition of genomic data and clinical information

We downloaded the expression of transcriptome profiling and clinical files of NB patients from the TARGET and GEO (gene ID: GSE49710) databases, which were used as the training set and validation sets, respectively. Samples without clinical data and those with less than 30 days of follow-up were excluded. We converted the probes to Gene Symbol with one probe corresponding to multiple genes. Additionally, we used the median value for the expression of multiple Gene Symbols. Finally, 144 and 498 tumour samples were obtained from the TARGET and GEO databases, respectively. Considering the batch effects, the R package “SVA” was utilised to reduce batch-to-batch variation in different datasets (13).

### Nonnegative matrix factorisation (NMF) clustering for FRGs

To identify distinct ferroptosis patterns, 249 validated FRGs were extracted from the FerrDb website. The FRGs are summarised in Supplementary Table S1. NMF clustering algorithms were used to categorise patients and discover distinct ferroptosis patterns based on the expression of 249 FRGs. We performed PCA to reduce dimensionality and determine the ability to distinguish patients. Kaplan-Meier survival curves were plotted to assess the OS between distinct subtypes. The relationships between different subtypes and clinicopathological features were further assessed. In addition, the level of 28 immune cell infiltration in the TME was quantified using the ssGSEA algorithm and compared between the two clusters.

### Functional enrichment analysis

We investigated the activity of biological pathways between distinct ferroptosis patterns using GSVA. With the “limma” package in R, differentially expressed genes (DEGs) (|fold change| > 1.5 and adjust *p* < 0.05) between different clusters were screened out and applied to GO and KEGG pathway analyses using the “clusterProfiler” R package.

### Construction of ferroptosis-related prognostic signature

The FRGs were further analysed by univariate Cox regression assessment using the Kaplan-Meier “survival” R package to screen those that correlated with the OS of NB. The prognosis-related FRGs were brought into LASSO Cox regression with R package “glmnet”, and we established a prognostic signature (risk score) based on the genes with non-zero regression coefficients. The risk score was obtained by multiplying gene expression by the LASSO regression coefficient, and patients with NB were then split into low- and high-risk subgroups based on the median risk score. The risk score for each patient in the GSE49710 dataset was calculated using the same coefficients and normalised expression microarray data for NB. We used the R packages “survminer” and “survival” to analyse the OS difference between the two risk groups. To evaluate the predictive performance of the risk score, the R package “survivalROC” was used to draw time-dependent receiver operating characteristic (ROC) curves. Principal component analysis (PCA) using the “scatterplot3d” package in R software was used to better differentiate NB patients into two risk score subgroups. The performance of the signature was evaluated using the restricted mean survival (RMS) curve.

### Clinical value of the risk score

To further confirm the clinical role of the risk score, the relationship between the risk score and the clinicopathological parameters was explored, including age, sex, MYCN status, INSS stage, histology, grade, mitosis-karyorrhexis index (MKI), and COG risk. Next, independent risk factors were screened using univariate and multivariate Cox regression analyses. We further performed Kaplan–Meier survival analysis in a training set with different clinical subgroups and explored the discrepancies between the two groups.

### Analysis of immune cell infiltration and TME

The R package “estimate” was used to calculate the stromal, immune, and estimated scores in the training set to evaluate the TME and further explore the differences between the two TME subgroups. We then explored the infiltration levels of various TIICs in NB by the CIBERSORT algorithm and compared the relative expression levels of 22 immune cells in different groups.

### Quantification of immune response predictor

The TIDE algorithm was used to predict cancer immunotherapy response and tumour immune escape (14). Tumour cells with higher TIDE scores were more likely to trigger immune escape, suggesting a reduced response rate to immune checkpoint blockade therapy. We explored the differences in TIDE scores between the two risk groups. Additionally, the Wilcoxon rank-sum test was used to analyse gene expression at the immune checkpoints.

### Statistical analysis

All analyses and figures were completed using R 4.2.1. Unless otherwise stated, *p* < 0.05 indicates statistical significance.

## Results

### Identification of neuroblastoma subtypes based on FRGs

**Figure 1:**
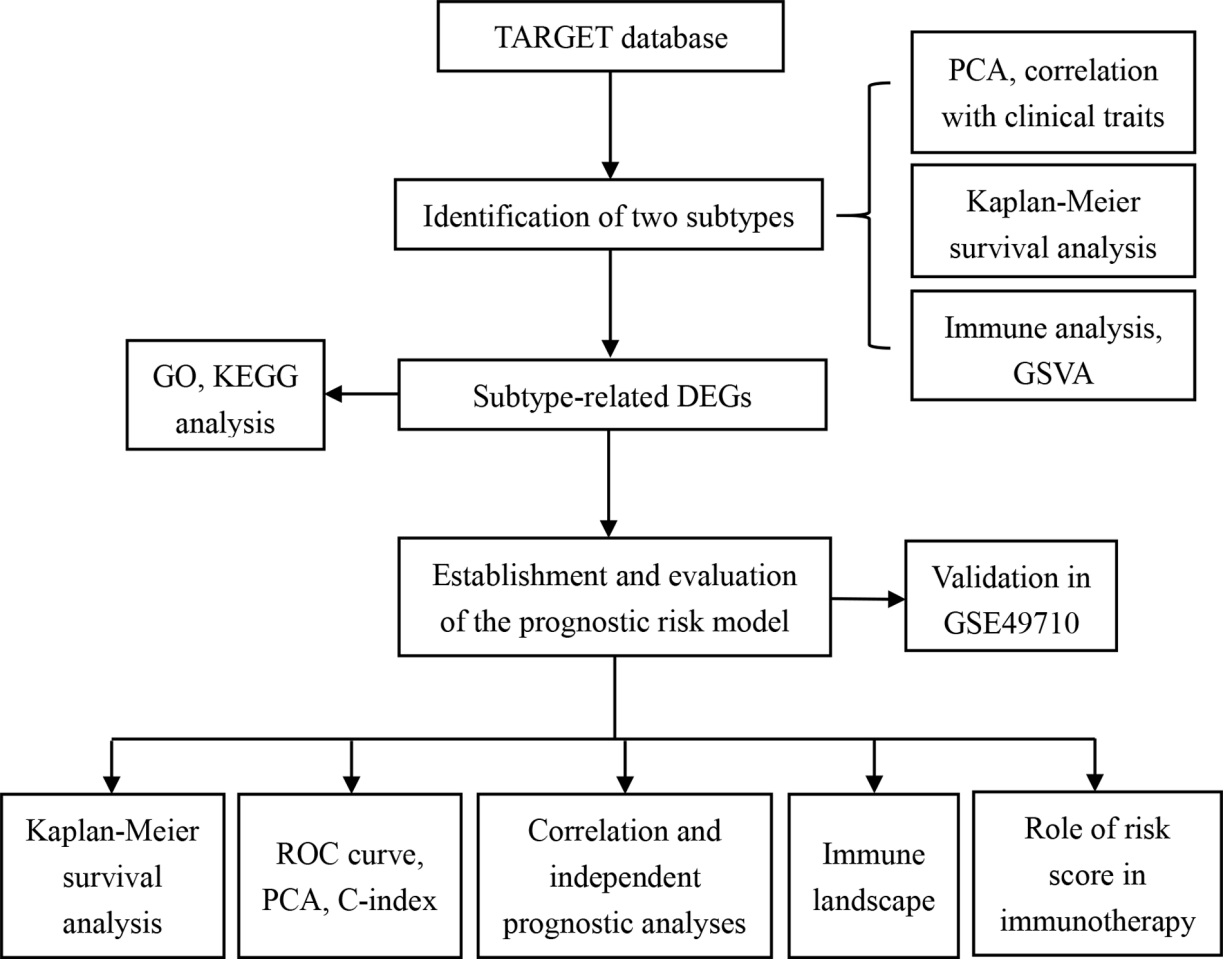
The flowchart of this study.

**Figure 2:**
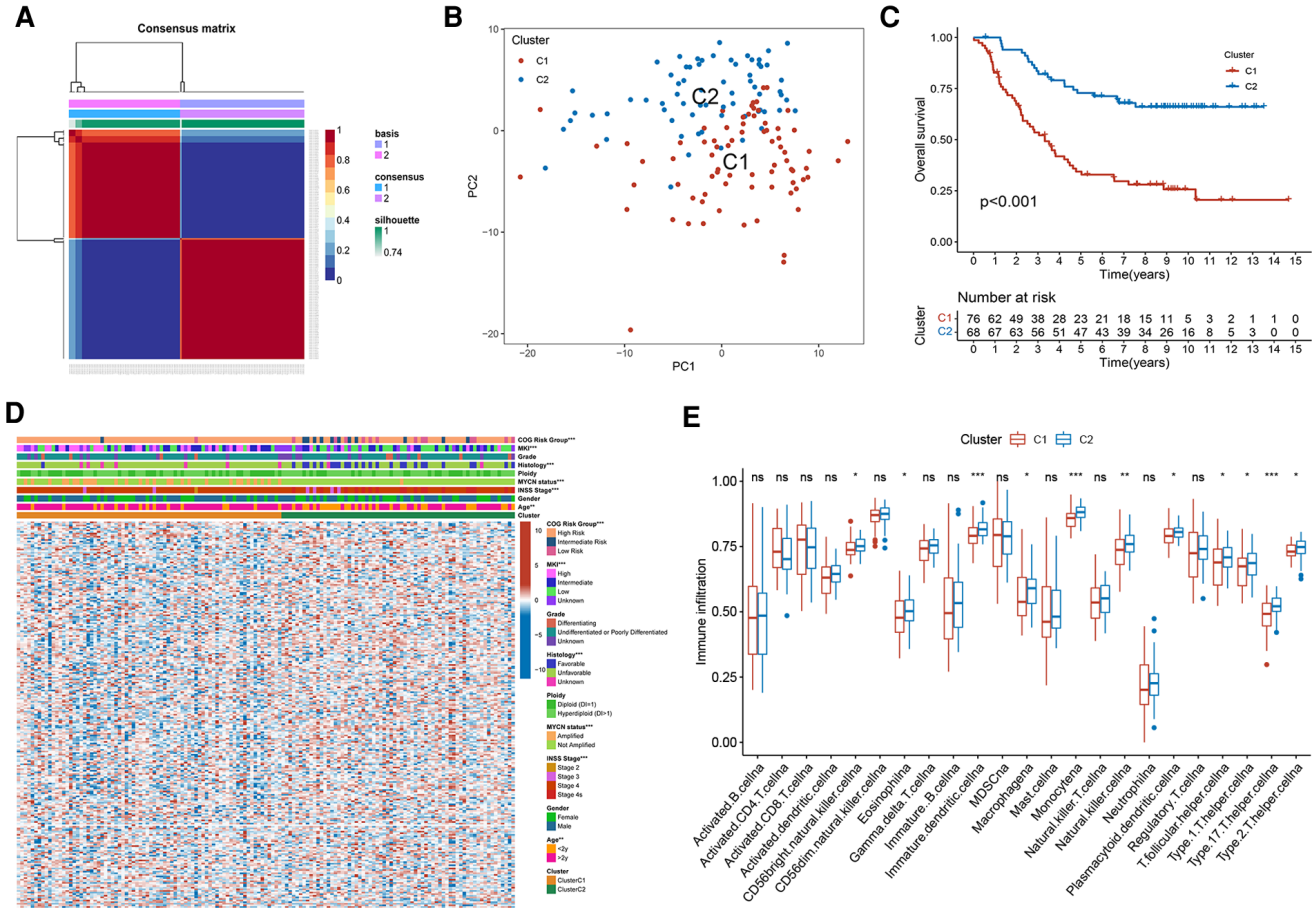
Two distinct ferroptosis subtypes were obtained from the training set based on FRGs. (**A**) The NMF clustering results when consensus matrix, *k* = 2. (**B**) The PCA scatter plot of two subtypes. (**C**) Kaplan-Meier curves of two ferroptosis subtypes in 144 patients from the training set. Log-rank test, *p* < 0.001. (**D**) Heatmap of the differences in FRGs expression and clinicopathological parameters between the two subtypes. (**E**) The abundance of different immune infiltrating cells in different subtypes.

This study progressed as per the workflow shown in the flowchart (Figure 1). In total, 249 validated FRGs were retrieved from the FerrDb website. After intersecting with the expression profiles of the training set, 241 FRGs remained. The NMF clustering analysis was performed in a training set based on overlapping FRGs, and two independent ferroptosis subtypes were determined (Figure 2A). The PCA results indicated that the samples in the two NB clusters were well separated, suggesting that they were effectively distinguishable (Figure 2B). Among the two ferroptosis subtypes, cluster 2 had a significantly better prognosis than cluster 1 (*p* < 0.001; Figure 2C). Most clinicopathological parameters were significantly different between the two subtypes (Figure 2D). We subsequently explored the relative abundance of 28 immune-infiltrating cells between the different ferroptosis subtypes. As shown in Figure 2E, immature dendritic cells, CD56 (bright) natural killer (NK) cells, eosinophils, macrophages, monocytes, NK cells, plasmacytoid dendritic cells, T follicular helper cells, T helper type 1 (Th1) cells, Th2 cells, and Th 17 cells significantly infiltrated at a low level in cluster C1 compared to cluster C2.

### Functional enrichment analysis

**Figure 3:**
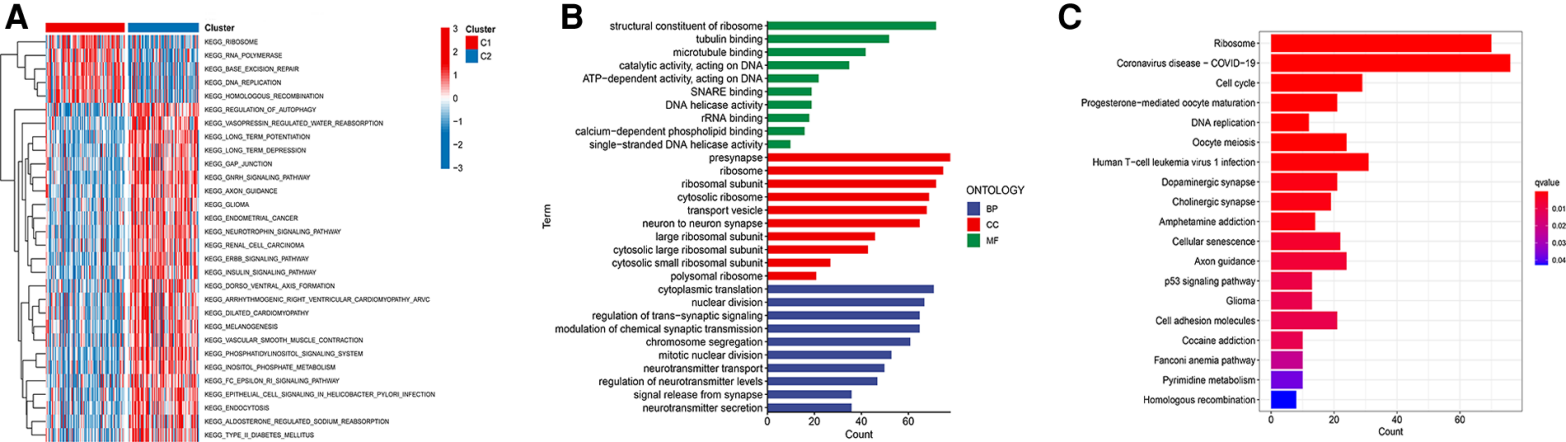
Functional enrichment analysis. (**A**) GSVA analysis of two ferroptosis subtypes in relatively activated hallmark gene sets. (**B**) Functional annotation of subtype-related DEGs by GO. (**C**) Functional annotation of subtype-related DEGs by KEGG.

For an in-depth knowledge of the biological pathways in the different ferroptosis patterns, GSVA analysis was performed between the two ferroptosis clusters, and the enrichment score was visualised (Figure 3A). Cluster C1 was significantly enriched in pathways related to genetic information processing, replication, and repair, such as ribosomes, RNA polymerase, and base excision repair. In contrast, cluster C2 was markedly enhanced in signal transduction pathways, as exhibited by processes related to the regulation of autophagy (Figure 3A). We further discovered 1,383 differentially expressed genes (DEGs) associated with the ferroptosis phenotype and carried out functional enrichment analysis to explore the potential biological function of the ferroptosis pattern. The first 10 pathways enriched in the three functional categories (BP, CC, and MF) are displayed in bubble diagrams (Figure 3B). KEGG pathway analysis revealed that FRGs participated in many important pathways related to tumour and genetic information processing, including the cell cycle, p53 signalling pathway, ribosome, and DNA replication (Figure 3C).

### Establishment of a prognostic risk model based on FRGs

**Figure 4:**
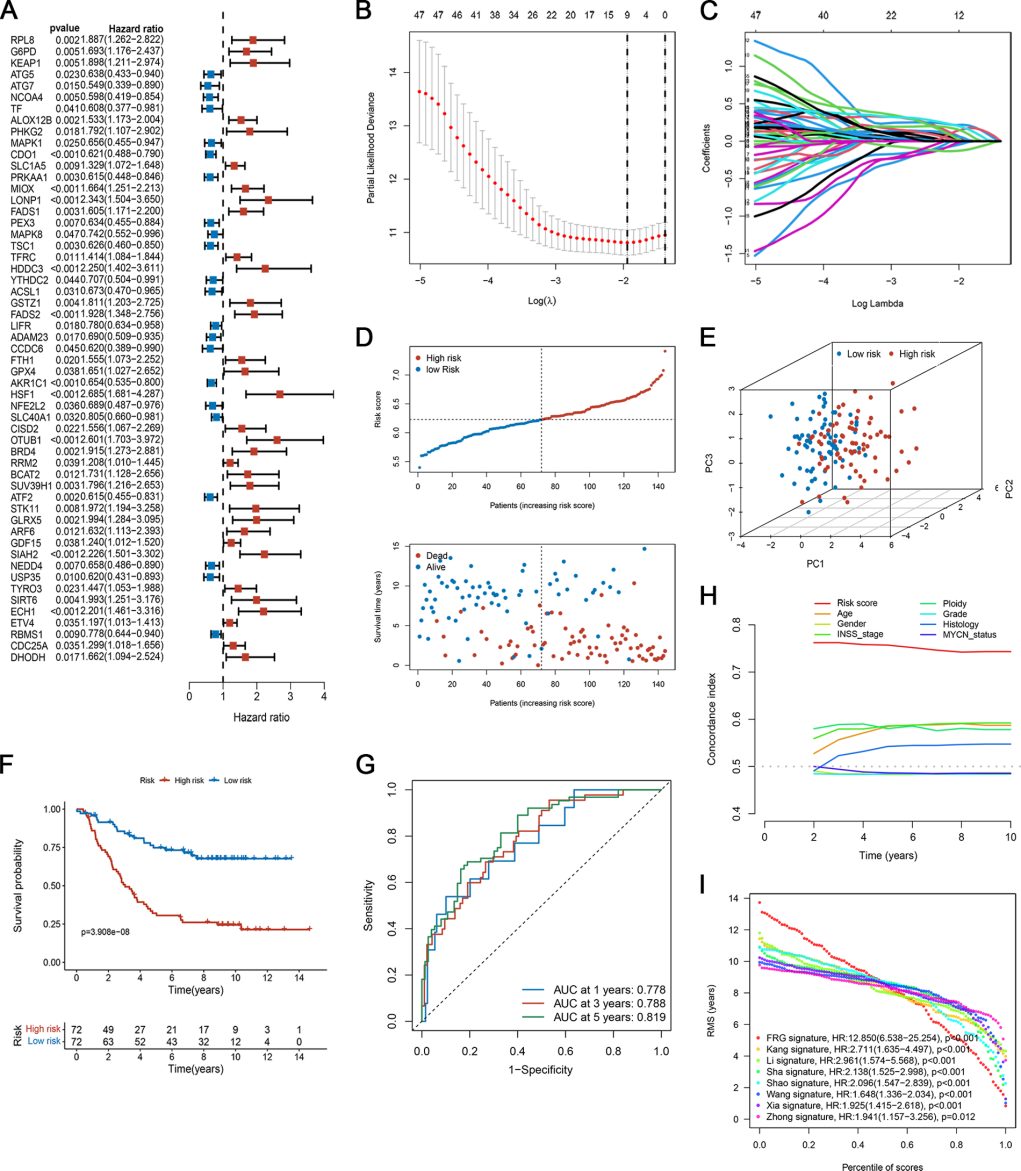
Development of the ferroptosis-based prognostic model in the training set. (**A**) Forest plot of FRGs related to overall survival, analyzed by univariate Cox regression. (**B**) Plots of the ten-fold cross-validation error rates. (**C**) The spectrum of LASSO coefficients of the nine FRGs. (**D**) The survival distribution of patients in the training focus increased patient mortality with the increase in risk score. (**E**) PCA showed remarkable differences between the two risk groups. (**F**) Kaplan-Meier survival curve between the two risk subgroups. (**G**) TdROC curve based on risk scores. (**H**) C-index of the risk score and clinical traits. (**I**) RMS curves for the composite prognostic signature and other clinical models.

By combining FRGs expression profiling with clinical follow-up data, 144 NB specimens were screened in the training set. To determine the role of FRGs in the evaluation of OS of neuroblastoma patients, we screened seven prognostic-related FRGs with significant differences using univariate Cox analysis (Figure 4A). Subsequently, we developed a prognostic risk model based on these nine FRGs. The regression coefficient of each gene was estimated by applying LASSO Cox regression, and the optimal lambda (*λ*) was derived by ten-fold cross-validation (Figures 4B,C).

The risk score of each patient was calculated as follows:

Risk score = (−0.03256 × CDO1exp) + (0.00819 × MIOXexp) + (0.03947 × HDDC3exp) + (0.04813 × FADS2exp) + (−0.12234 × AKR1C1exp) + (0.21927 × HSF1exp) + (0.15846 × OTUB1exp) + (0.18614 × SIAH2exp) + (0.13281 × ECH1exp)

### Evaluation of the prognostic risk model

The patients were divided into high- and low-risk groups based on the median risk score. Figure 4D shows the distribution of risk scores and survival status of patients with NB. Patients in the high-risk group had an elevated risk score and a worse prognosis. The PCA results showed that the samples in the two risk groups could be clearly distinguished (Figure 4E). As suggested by the Kaplan-Meier curves, low-risk patients shared significantly superior OS to high-risk patients (Figure 4F). Also, the ROC curves in Figure 4G, the AUC at 1-, 3-, and 5- years was 0.778, 0.788, and 0.819, respectively, suggesting that the risk scores have a good ability to predict OS. We compared the C-index of the risk score with that of several clinical parameters. The predictive accuracy of the prognostic model was significantly better than that of other clinical features (Figure 4H). Furthermore, we compared our proposed model with previously published NB models. Our signature had a clear advantage over previously published models for assessing prognosis (Figure 4I; Supplementary Figure S1).

**Figure 5:**
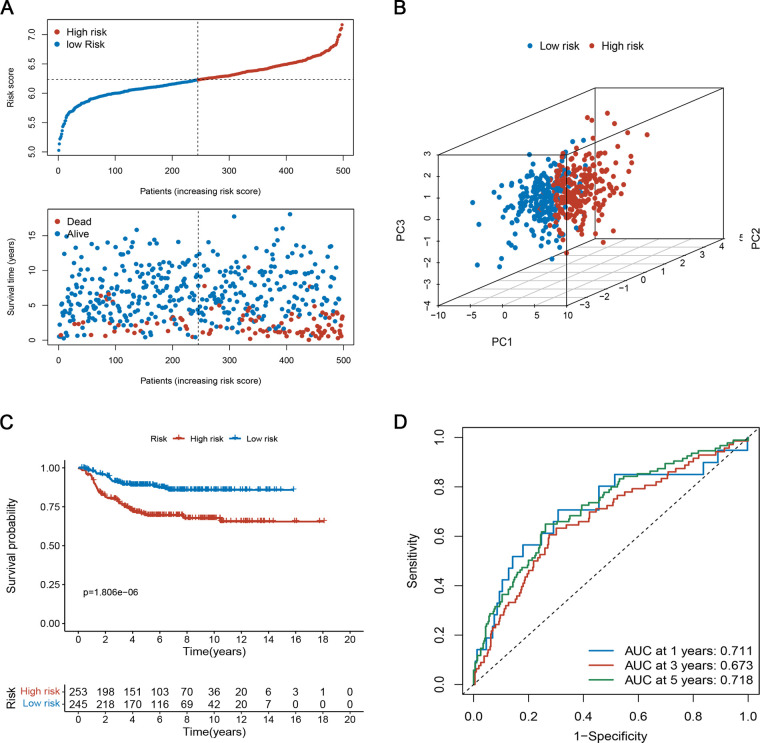
Evaluation of the prognostic risk model in the testing set. (**A**) The survival distribution of patients in the training focus increased patient mortality with the increase in risk score. (**B**) PCA showed remarkable differences between the two risk groups. (**C**) Kaplan-Meier survival curve between the high- and low-risk groups. (**D**) Time-dependent ROC curve based on risk scores.

To assess the robustness of the prognostic signature in predicting OS of NB, we used the same risk model formula for verification in the verification set. The results were similar (Figures 5A–D). As shown in Figure 5A, the high-risk group had a higher proportion of deaths. Again, PCA demonstrated the reliable clustering ability of the risk score (Figure 5B). Kaplan-Meier survival analysis indicated that the prognosis of the high-risk group was poor (*p* < 0.001, Figure 5C). The 1-, 3-, and 5- year's AUC values were 0.711, 0.673, and 0.718, respectively (Figure 5D).

### The relationship between risk scores and clinical characteristics

**Figure 6:**
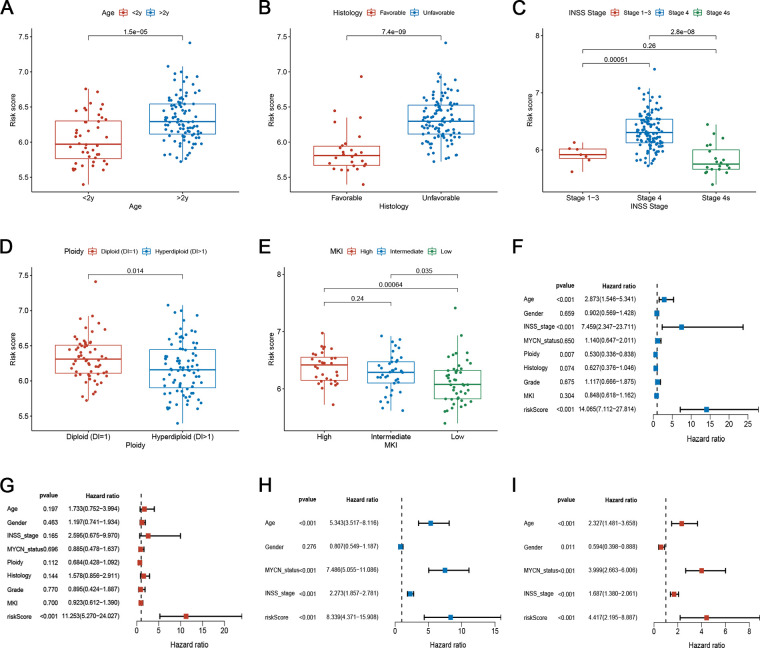
Correlation between risk scores and clinical characteristics. (**A**–**E**) Correlation between risk score and various clinicopathological parameters in the training set. The age, histology, INSS stage, Ploidy, and MKI were used as sample annotations. (**F-G**) Univariate and multivariate Cox analyses of risk scores and clinical traits in the training set. (**H–I**) Univariate and multivariate Cox analyses of risk scores and clinical features in the testing set.

We examined the relationship between risk scores and clinical characteristics in the TARGET set and found that the risk score was significantly higher in the >2 years old group, unfavourable histology group, advanced INSS stage group, diploid (DI = 1) group, and high MKI group (Figures 6A–E). To determine whether the risk score of the model could independently indicate the prognosis of patients with NB, we included patients with NB in the Cox regression analysis (Figures 6F–I). In both the TARGET and GEO datasets, multi-factor Cox analysis suggested that the risk score was an independent predictive factor for evaluating individual OS (Figures 6G,I). Stratified analysis was then conducted for different clinical subgroups. The survival curves in Supplementary Figure S2 show that the risk scores still had high-risk stratification ability in the different subgroups. These results indicate that low-risk patients shared significantly superior OS to high-risk patients. The above results indicate that the risk model might be a prognostic biomarker that could effectively predict overall survival and some of the clinical features in NB patients.

### Differences in the immune landscape between different risk groups

**Figure 7:**
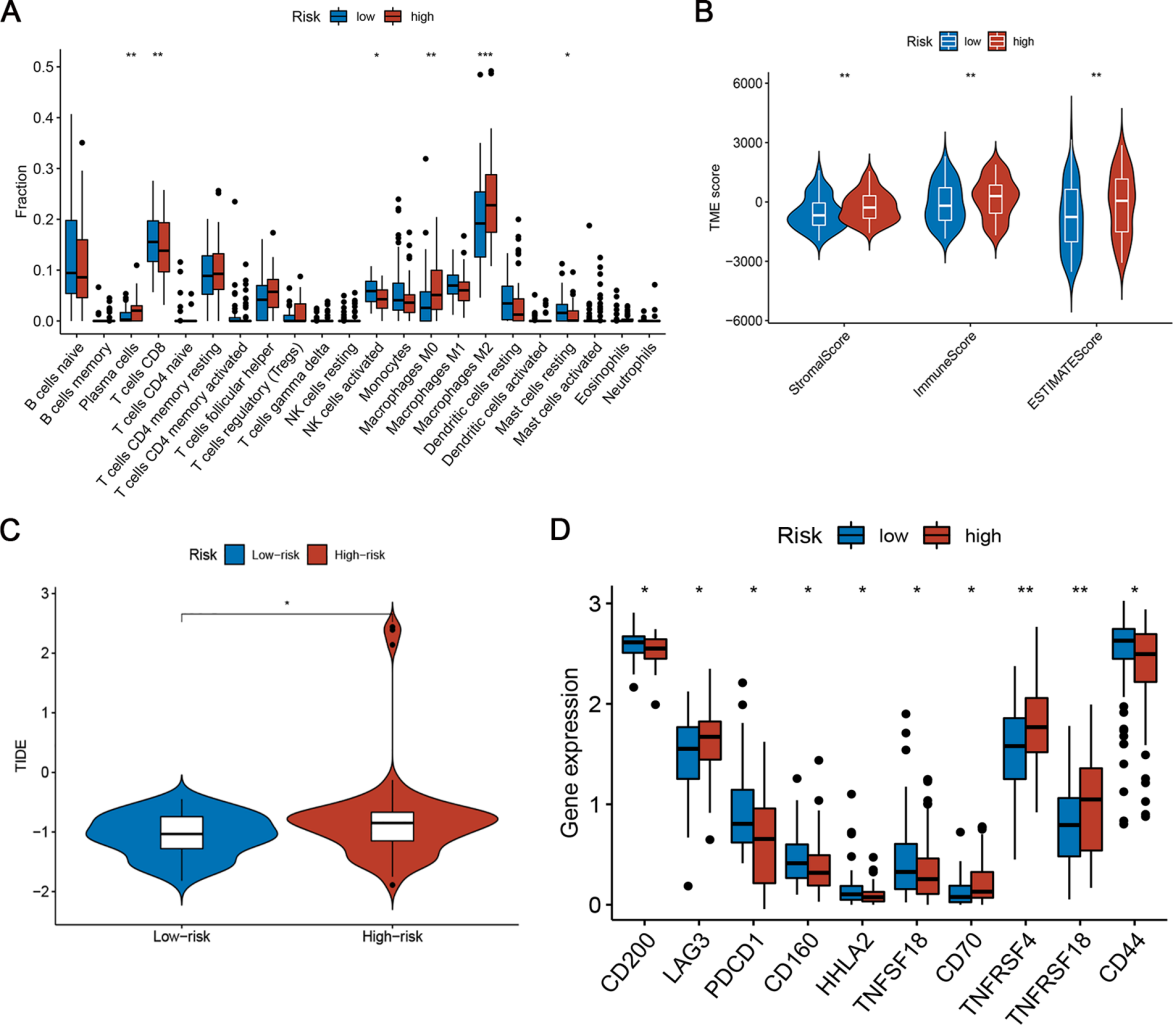
Differences in TIME characteristics and immunotherapeutic benefits between different risk groups. (**A**) Comparison of immune infiltrating cells between low- and high-risk subgroups. (**B**) Immunity and stromal infiltration scores for the two risk subgroups. (**C**) Boxplot of TIDE for different risk score groups. (**D**) Analysis of immune checkpoint-related genes in the low- and high-risk subgroups.

To explore the relationship between risk scores and immune status, we explored the infiltration state of 22 types of TIICs in the TARGET set. The results showed that the infiltration levels of CD8+ T cells, activated NK cells, and resting mast cells in the high-risk group were significantly lower than those in the low-risk group (Figure 7A). In contrast, the infiltration of plasma cells and M0 and M2 macrophages was higher in the high-risk group (Figure 7A). Furthermore, the numbers of CD8+ T cells, CD11b + NK cells, and CD206 + M2 macrophages in resected NB specimens were evaluated using immunohistochemistry. Consistent with the results of bioinformation analysis, the infiltration levels of CD8+ T cells and CD11b + NK cells were significantly decreased in high-risk patients, while the infiltration levels of CD163 + and CD206+ M2 macrophages were increased (Supplementary Figure S3). Figure 7B shows the tumour stromal and immune scores calculated by the ESTIMATE algorithm. In the high-risk group, the fraction of stromal cells and the infiltration of immune cells was higher. To explore the predictive ability of risk scores for the benefit of immunotherapy, we calculated the TIDE score of samples and compared the TIDE scores of the two risk subgroups. The TIDE score substantially decreased in the low-risk subgroup (Figure 7C), indicating that the low-risk subgroup was more likely to benefit from immunotherapy. We also compared the expression of 34 immune checkpoint-related genes in the two risk groups and found that they were differentially expressed among two groups (Figure 7D). Thus, the ferroptosis-related risk model may indicate a response to immunotherapy in NB.

## Discussion

Neuroblastoma is the most common extracranial tumour in children and accounts for 15% of all childhood cancer-related deaths (2). Neuroblastomas are characterised by a high degree of heterogeneity, which results in a highly variable prognosis for patients and limits the efficacy of the existing treatment modalities (15). Although patients with low- and moderate-risk NB usually have a good prognosis after surgery, treatment options for high-risk patients are very limited (16). Ferroptosis is a new pattern of RCD with strong antitumour potential (17). It is an iron-dependent form of cell death that is molecularly, morphologically, and biochemically distinct from the other types of cell death (16, 18). Ferroptosis can be induced pharmacologically and is expected to be useful in translational medicine. Studies have shown that the use of classical or non-classical iron deposition inducers or combination therapy is a potential treatment for high-risk NB (19, 20). For example, withaferin A has been shown to eradicate high-risk NB by inducing ferroptosis *via* canonical pathways, implicating a direct target of GPX4 (19). Therefore, identifying distinct ferroptosis patterns in NB will help us to better understand the biological variation of the condition, screen effective prognostic markers, and implement more effective treatment regimens.

To investigate the functions and mechanisms of ferroptosis in the progression of NB, we divided the samples into two subtypes, C1 and C2. This was based on the expression of FRGs with distinct biological behaviours and clinical outcomes. Most FRGs were downregulated in cluster C1, and patients in cluster C1 had a worse prognosis and advanced clinicopathological features. This indicated that the downregulation of FRGs may contribute to the progression of NB. The prognostic difference between the two subtypes of samples may be determined by the differences in biological functions and signalling pathways, as well as the difference in the infiltration level of TIICs between the two ferroptosis subtypes. Pathways enriched in C1 seem to be closely related to the biological processes of genetic information processing, replication, and repair. Most of the enrichments were related to the signal transduction pathways in C2. We also found that samples in C1 had low infiltration levels of NK cells, dendritic cells, macrophages, monocytes, eosinophils, Th1 cells, Th2 cells, and Th 17 cells. These were also found to be especially low in high-risk NB and were associated with a favourable prognosis (4, 21, 22), which is consistent with the results of our study. Taken together, ferroptosis subtypes may be a valuable tool for predicting the clinical outcomes of NB patients.

We further established a ferroptosis signature to quantify the individual risk scores and validated the results in an independent set. The risk score model contained nine signature genes, including CDO1, MIOX, HDDC3, FADS2, AKR1C1, HSF1, OTUB1, SIAH2, and ECH1. Of all these, CDO1, a cancer-specific methylated gene, has been shown to play a cancer suppressor role in various cancers (23, 24). CDO1 is also a non-haeme iron enzyme that ultimately leads to elevated ROS levels and induction of ferroptosis by inhibiting the production of glutathione from cysteine (25). Hao et al*.* (26) found that silencing of CDO1 suppressed erastin-induced ferroptosis in gastric cancer cells both *in vitro* and *in vivo* by upregulating the expression of GPX4. Another gene, FADS2 balances lipid metabolic activity and redox-driven ferroptosis in hydrophobic ovarian cancer cells by regulating the GSH/GSSG ratio and GPX4 (27). Studies have shown that HSF1 regulates multiple types of cell death through different signalling pathways (28, 29). The ubiquitin hydrolase OTUB1 is a key regulator of ferroptosis and its expression is upregulated in tumours (30, 31). Zhao et al. (32) indicated that OTUB1 inhibits ferroptosis and promotes stemness of glioma cells by stabilising the SLC7A11 protein. In conclusion, genes that drive or inhibit cell ferroptosis have a significant influence on the occurrence, progression, and metastasis of tumours. Targeting these genes and inducing cell ferroptosis may be a novel therapeutic method to combat NB.

With the continuous exploration of the prognostic model, we are surprised that the risk model has great potential for guiding immunotherapy. The tumour immune microenvironment (TIME) comprises the structure, functional metabolism, stromal cells, immune cells, and tumour cells of the tissue in which the tumour is located (33, 34). TIME has a tremendous influence on tumourigenesis, progression, and immunotherapeutic response (35, 36). Tumours choose cancer cells with lower immunogenicity to escape the immune system. This may cause an increase in immunosuppressive cells and a decrease in immune response cells (37). Simultaneously, the microenvironment for tumour growth can serve as a “fertile soil” for cancer cells to promote tumour growth and development (38). We observed that the risk score was negatively associated with the abundance of activated NK cells, CD8+ T cells, and resting mast cells, and positively correlated with the abundance of plasma cells, M0 and M2 macrophages. We considered that FRGs in NB tissue can lead to an imbalance in the proportion of immune cell infiltration and thereby affect the effect of blocking PD-1/PD-L1. Wang et al*.* revealed that activated CD8+ T cells significantly increase lipid peroxidation and make tumour cells more prone to ferroptosis (39). Depending on the different microenvironments of various tissues, macrophages can polarise into M1 or M2 subtypes (40). Tumour-associated macrophages (TAMs) have been shown to predict adverse clinical outcomes in NB, and M2-phenotypic TAMs contain a population of immune cells that promote tumour metastasis, contribute to immune suppression, and lead to the failure of checkpoint inhibitor therapies (41). Consistent with previous studies, the infiltration abundance of M2 macrophages was evidently higher in the high-risk subgroup with worse prognosis in this study. Thus far, immunotherapy has emerged as a new treatment strategy for cancer. We found that the TIDE score was substantially decreased in the low-risk group, indicating that patients with low-risk scores might benefit from immunotherapy. When performing immune checkpoint analysis, we observed significant differences in some immune checkpoints between the high- and the low-risk groups, which provided more and better options for NB patients to receive immune checkpoint inhibitor (ICIs) treatment.

## Conclusions

We comprehensively evaluated 241 FRGs and defined two distinct molecular subtypes with different clinical outcomes and infiltrating immune cells. We further developed a novel risk model for neuroblastoma patients based on nine FRGs and systematically explored the relationship between the risk score and TME cell-infiltrating characteristics. This may aid in the use of ferroptosis parameters to enhance our recognition of TME characteristics and provide guidance for immunotherapy in neuroblastoma patients.

## Data Availability

Publicly available datasets were analyzed in this study. This data can be found here: The datasets presented in this study can be found in TARGET databases (https://ocg.cancer.gov/programs/target/data-matrix) and GEO (gene ID: GSE49710; https://www.ncbi.nlm.nih.gov/geo/).
