# Supplementary figures and images for: Development of A novel ferroptosis-related prognostic signature with multiple significance in paediatric neuroblastoma
Source: Front Pediatr. 2023 Feb 22;11:1067187. doi: 10.3389/fped.2023.1067187 (PMC9992189; doi:10.3389/fped.2023.1067187)

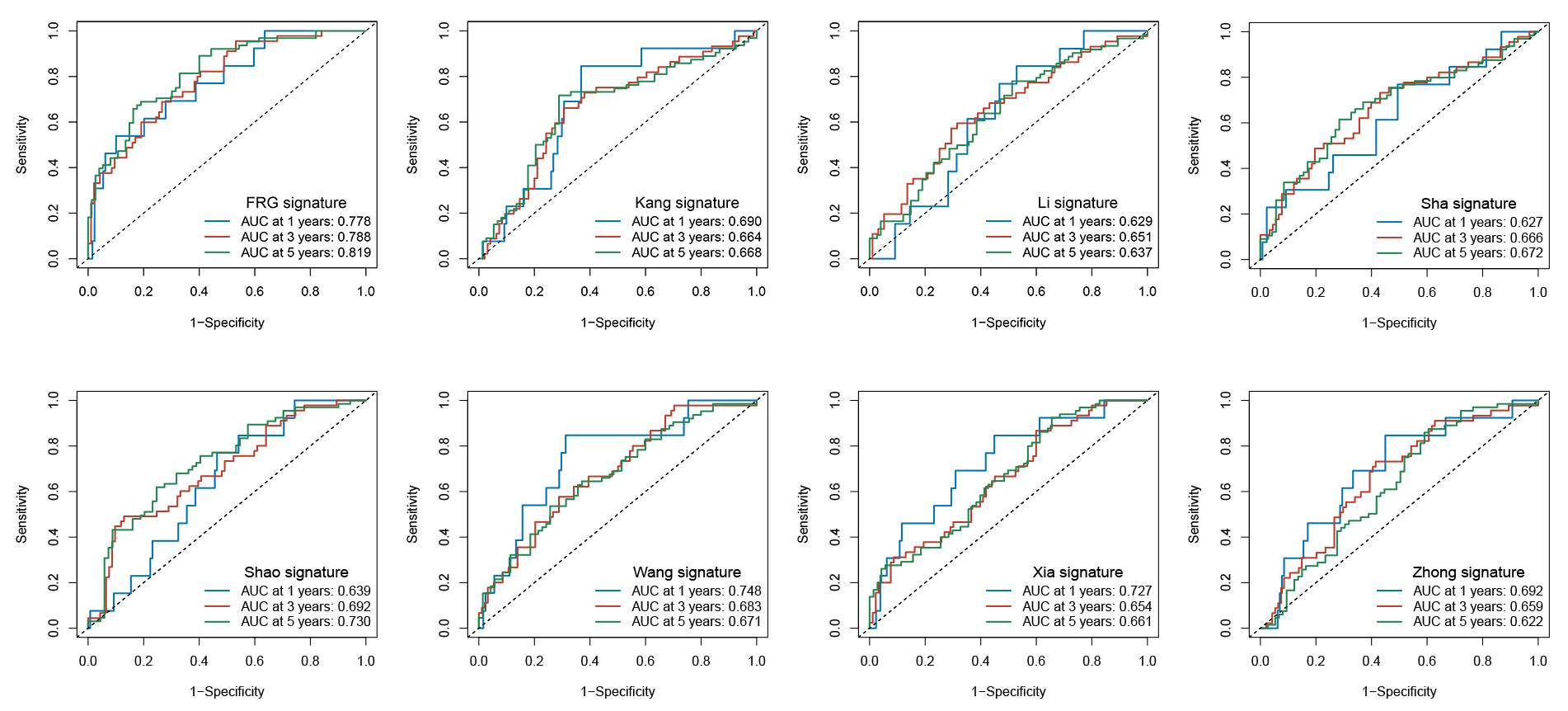

Supplement: Supplementary file 1 [file Image1.tif]

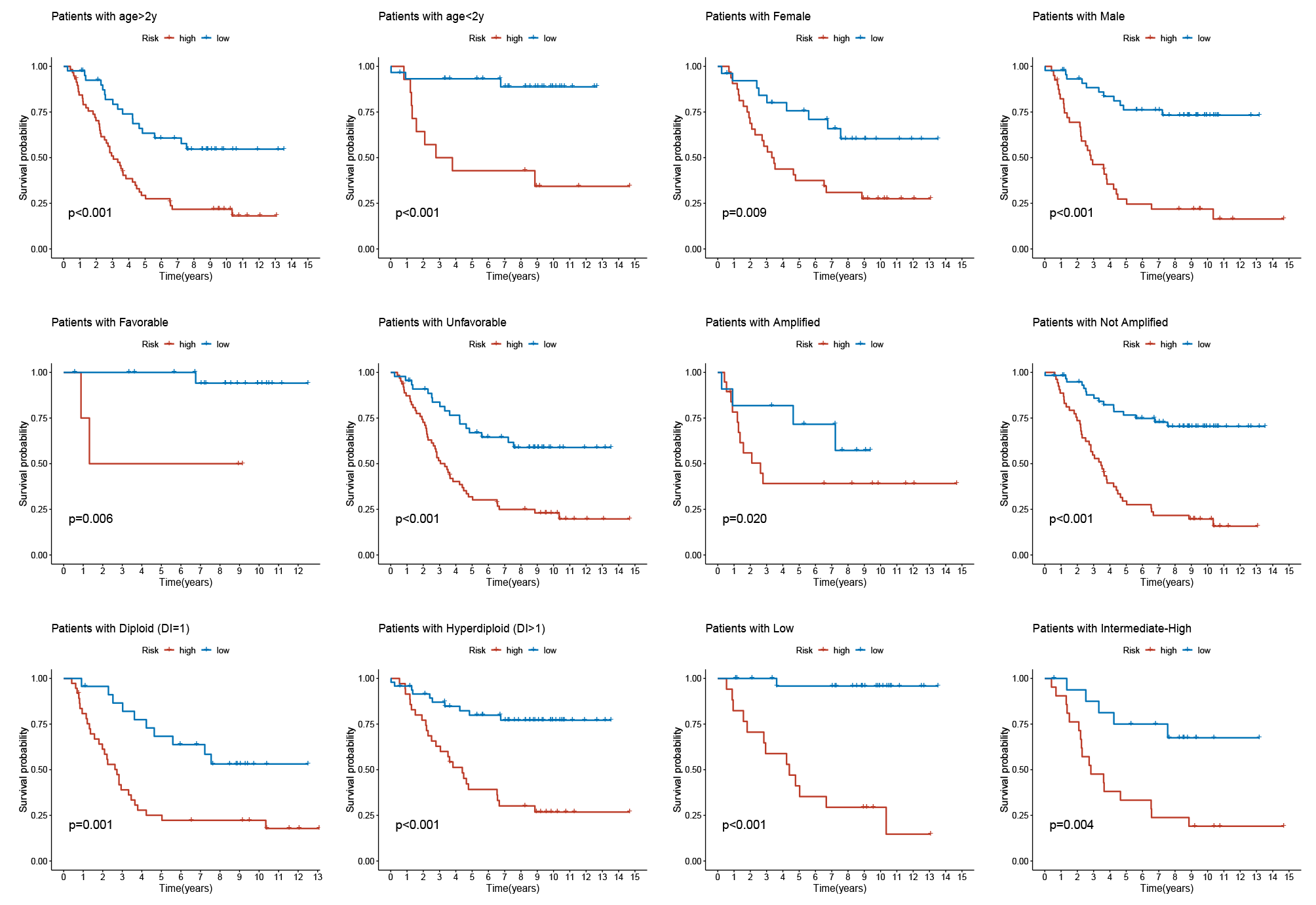

Supplement: Supplementary file 2 [file Image2.tif]

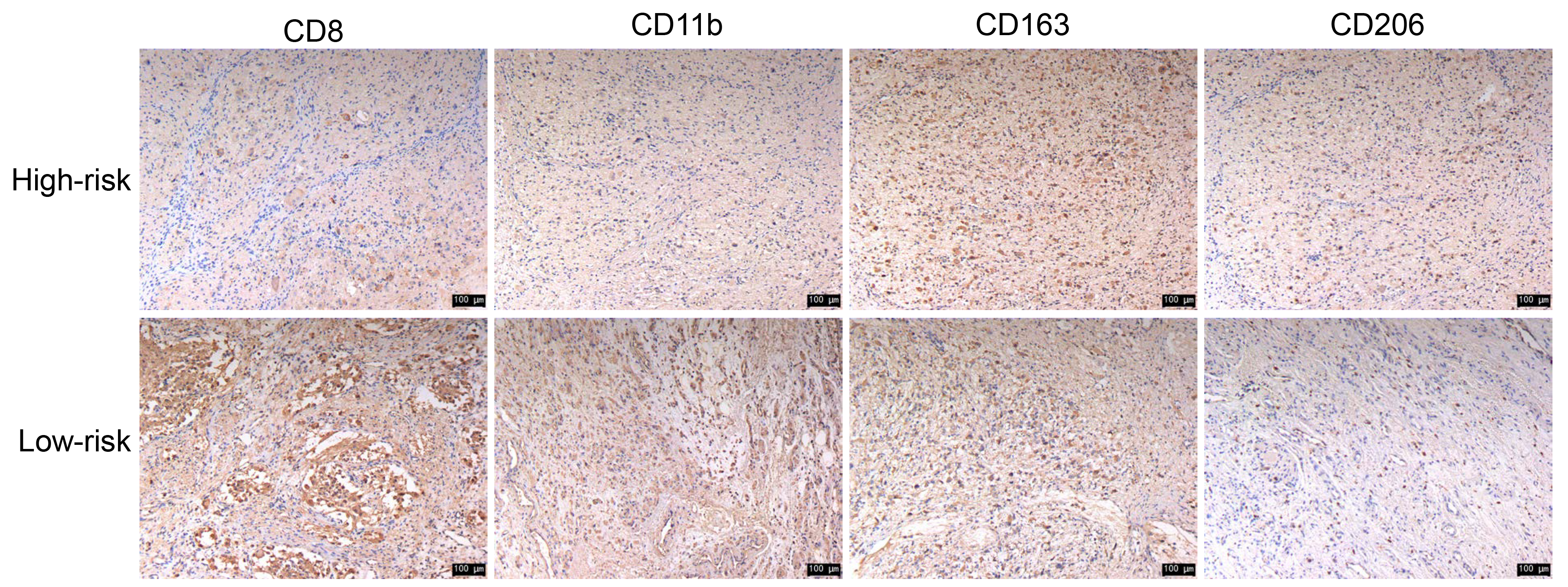

Supplement: Supplementary file 3 [file Image3.tif]
