# Supplementary material for: Development of A novel ferroptosis-related prognostic signature with multiple significance in paediatric neuroblastoma
Source: Front Pediatr. 2023 Feb 22;11:1067187. doi: 10.3389/fped.2023.1067187 (PMC9992189; doi:10.3389/fped.2023.1067187)
Supplement: Supplementary file 4 [file Table1.docx]

**Table S1 The list of 249 validated ferroptosis-related genes.**

| symbol | confidence | testin | uniformgenetype |
| --- | --- | --- | --- |
| RPL8 | Validated | Human | gene with protein product |
| IREB2 | Validated | Human | gene with protein product |
| ATP5MC3 | Validated | Human | gene with protein product |
| CS | Validated | Human | gene with protein product |
| EMC2 | Validated | Human | gene with protein product |
| ACSF2 | Validated | Human | gene with protein product |
| G6PD | Validated | Human | gene with protein product |
| PGD | Validated | Human | gene with protein product |
| VDAC2 | Validated | Human | gene with protein product |
| TP53 | Validated | Human, mice | gene with protein product |
| CARS1 | Validated | Human, rat | gene with protein product |
| KEAP1 | Validated | Human, mice | gene with protein product |
| HMOX1 | Validated | Human, mice | gene with protein product |
| ATG5 | Validated | Human, mice | gene with protein product |
| ATG7 | Validated | Human, mice | gene with protein product |
| NCOA4 | Validated | Human | gene with protein product |
| TF | Validated | Human | gene with protein product |
| ALOX5 | Validated | Human | gene with protein product |
| ALOX12 | Validated | Human | gene with protein product |
| ALOX12B | Validated | Human | gene with protein product |
| ALOX15 | Validated | Human | gene with protein product |
| ALOX15B | Validated | Human | gene with protein product |
| ALOXE3 | Validated | Human | gene with protein product |
| PHKG2 | Validated | Human | gene with protein product |
| ACSL4 | Validated | Human | gene with protein product |
| SAT1 | Validated | Human, mice | gene with protein product |
| EGFR | Validated | Human | gene with protein product |
| NOX4 | Validated | Human | gene with protein product |
| MAPK3 | Validated | Human | gene with protein product |
| MAPK1 | Validated | Human | gene with protein product |
| ZEB1 | Validated | Human | gene with protein product |
| DPP4 | Validated | Human, mice | gene with protein product |
| CDKN2A | Validated | Human, mice | gene with protein product |
| PEBP1 | Validated | Human, mice | gene with protein product |
| SOCS1 | Validated | Human | gene with protein product |
| CDO1 | Validated | Human | gene with protein product |
| MYB | Validated | Human | gene with protein product |
| SLC1A5 | Validated | Human | gene with protein product |
| CHAC1 | Validated | Human | gene with protein product |
| GOT1 | Validated | Human | gene with protein product |
| BECN1 | Validated | Human, mice | gene with protein product |
| PRKAA2 | Validated | Human | gene with protein product |
| PRKAA1 | Validated | Human | gene with protein product |
| ELAVL1 | Validated | Human, mice | gene with protein product |
| BAP1 | Validated | Human | gene with protein product |
| ABCC1 | Validated | Human | gene with protein product |
| ACVR1B | Validated | Human | gene with protein product |
| TGFBR1 | Validated | Human | gene with protein product |
| IFNG | Validated | Human, mice | gene with protein product |
| ANO6 | Validated | Human, mice | gene with protein product |
| HMGB1 | Validated | Human, mice | gene with protein product |
| TNFAIP3 | Validated | Human | gene with protein product |
| ATF3 | Validated | Human | gene with protein product |
| ATM | Validated | Human | gene with protein product |
| YY1AP1 | Validated | Human, mice | gene with protein product |
| EGLN2 | Validated | Human, mice | gene with protein product |
| MIOX | Validated | Human, mice | gene with protein product |
| TAFAZZIN | Validated | Human, mice | gene with protein product |
| MTDH | Validated | Human, mice | gene with protein product |
| IDH1 | Validated | Human | gene with protein product |
| PANX1 | Validated | Human, mice | gene with protein product |
| LONP1 | Validated | Human | gene with protein product |
| CD82 | Validated | Human | gene with protein product |
| IL1B | Validated | Human, mice | gene with protein product |
| POR | Validated | Human, mice | gene with protein product |
| CYB5R1 | Validated | Human | gene with protein product |
| ELOVL5 | Validated | Human | gene with protein product |
| FADS1 | Validated | Human | gene with protein product |
| FBXW7 | Validated | Human | gene with protein product |
| IL6 | Validated | Human, mice | gene with protein product |
| ATF4 | Validated | Human, mice | gene with protein product |
| PEX10 | Validated | Human, mice | gene with protein product |
| PEX12 | Validated | Human | gene with protein product |
| AGPS | Validated | Human | gene with protein product |
| PEX2 | Validated | Human | gene with protein product |
| FAR1 | Validated | Human | gene with protein product |
| PEX3 | Validated | Human | gene with protein product |
| SIRT1 | Validated | Human, mice | gene with protein product |
| GSK3B | Validated | Human | gene with protein product |
| MAPK8 | Validated | Human | gene with protein product |
| BRD7 | Validated | Human, mice | gene with protein product |
| SLC25A28 | Validated | Human, mice | gene with protein product |
| SLC11A2 | Validated | Human | gene with protein product |
| TSC1 | Validated | Human, mice | gene with protein product |
| SNCA | Validated | Human | gene with protein product |
| SIRT3 | Validated | Human, porcine | gene with protein product |
| TFRC | Validated | Human | gene with protein product |
| CGAS | Validated | Human | gene with protein product |
| STING1 | Validated | Human | gene with protein product |
| HDDC3 | Validated | Human | gene with protein product |
| MDM2 | Validated | Human | gene with protein product |
| MDM4 | Validated | Human | gene with protein product |
| DLD | Validated | Human | gene with protein product |
| DNAJB6 | Validated | Human | gene with protein product |
| WWTR1 | Validated | Human | gene with protein product |
| PRKCA | Validated | Human | gene with protein product |
| SMPD1 | Validated | Human | gene with protein product |
| MYCN | Validated | Human | gene with protein product |
| SMG9 | Validated | Human | gene with protein product |
| TLR4 | Validated | Human, rat | gene with protein product |
| PAQR3 | Validated | Human | gene with protein product |
| MICU1 | Validated | Human | gene with protein product |
| QSOX1 | Validated | Human | gene with protein product |
| CLTRN | Validated | Human | gene with protein product |
| KLF2 | Validated | Human | gene with protein product |
| YTHDC2 | Validated | Human | gene with protein product |
| DDR2 | Validated | Human | gene with protein product |
| SLC39A7 | Validated | Human | gene with protein product |
| TRIM46 | Validated | Human | gene with protein product |
| ACSL1 | Validated | Human | gene with protein product |
| KDM5A | Validated | Human | gene with protein product |
| CYGB | Validated | Human | gene with protein product |
| GSTZ1 | Validated | Human | gene with protein product |
| ACO1 | Validated | Human | gene with protein product |
| GJA1 | Validated | Human, mice | gene with protein product |
| SLC7A11 | Validated | Human | gene with protein product |
| CIRBP | Validated | Human | gene with protein product |
| YAP1 | Validated | Human | gene with protein product |
| TRIM26 | Validated | Human | gene with protein product |
| FADS2 | Validated | Human | gene with protein product |
| PIEZO1 | Validated | Human, mice | gene with protein product |
| LIFR | Validated | Human | gene with protein product |
| PTPN6 | Validated | Human | gene with protein product |
| ADAM23 | Validated | Human | gene with protein product |
| CPEB1 | Validated | Human | gene with protein product |
| KDM6B | Validated | Human, mice | gene with protein product |
| METTL14 | Validated | Human, rat | gene with protein product |
| MIB1 | Validated | Human | gene with protein product |
| KDM5C | Validated | Human, mice | gene with protein product |
| CCDC6 | Validated | Human, mice | gene with protein product |
| PTGS2 | Validated | Human | gene with protein product |
| FTH1 | Validated | Human | gene with protein product |
| GPX4 | Validated | Human, mice | gene with protein product |
| AKR1C1 | Validated | Human | gene with protein product |
| AKR1C2 | Validated | Human | gene with protein product |
| AKR1C3 | Validated | Human | gene with protein product |
| RB1 | Validated | Human, mice | gene with protein product |
| HSPB1 | Validated | Human, mice | gene with protein product |
| HSF1 | Validated | Human, mice | gene with protein product |
| NFE2L2 | Validated | Human, mice | gene with protein product |
| SQSTM1 | Validated | Human, mice | gene with protein product |
| NQO1 | Validated | Human, mice | gene with protein product |
| MUC1 | Validated | Human | gene with protein product |
| MT1G | Validated | Human, mice | gene with protein product |
| SLC40A1 | Validated | Human | gene with protein product |
| CISD1 | Validated | Human | gene with protein product |
| HSPA5 | Validated | Human, mice | gene with protein product |
| HELLS | Validated | Human, mice | gene with protein product |
| SCD | Validated | Human | gene with protein product |
| SRC | Validated | Human | gene with protein product |
| STAT3 | Validated | Human | gene with protein product |
| PML | Validated | Human | gene with protein product |
| NFS1 | Validated | Human | gene with protein product |
| TP63 | Validated | Human | gene with protein product |
| CDKN1A | Validated | Human | gene with protein product |
| FH | Validated | Human | gene with protein product |
| CISD2 | Validated | Human | gene with protein product |
| CBS | Validated | Human, mice | gene with protein product |
| ISCU | Validated | Human | gene with protein product |
| ACSL3 | Validated | Human | gene with protein product |
| OTUB1 | Validated | Human, mice | gene with protein product |
| CD44 | Validated | Human | gene with protein product |
| BRD4 | Validated | Human | gene with protein product |
| PRDX6 | Validated | Human | gene with protein product |
| SESN2 | Validated | Human, mice | gene with protein product |
| NF2 | Validated | Human, mice | gene with protein product |
| ARNTL | Validated | Human, mice | gene with protein product |
| HIF1A | Validated | Human, mice | gene with protein product |
| CA9 | Validated | Human | gene with protein product |
| TMBIM4 | Validated | Human, mice | gene with protein product |
| AIFM2 | Validated | Human, mice | gene with protein product |
| LAMP2 | Validated | Human | gene with protein product |
| ZFP36 | Validated | Human, mice | gene with protein product |
| PROM2 | Validated | Human | gene with protein product |
| CHMP5 | Validated | Human, mice | gene with protein product |
| CHMP6 | Validated | Human, mice | gene with protein product |
| CAV1 | Validated | Human, mice | gene with protein product |
| GCH1 | Validated | Human | gene with protein product |
| PIR | Validated | Human, mice | gene with protein product |
| GCLC | Validated | Human | gene with protein product |
| HCAR1 | Validated | Human, mice | gene with protein product |
| SLC16A1 | Validated | Human, mice | gene with protein product |
| RRM2 | Validated | Human | gene with protein product |
| SREBF1 | Validated | Human | gene with protein product |
| SREBF2 | Validated | Human | gene with protein product |
| FZD7 | Validated | Human, mice | gene with protein product |
| BCAT2 | Validated | Human | gene with protein product |
| PLA2G6 | Validated | Human, mice | gene with protein product |
| PARK7 | Validated | Human, mice | gene with protein product |
| FXN | Validated | Human | gene with protein product |
| SUV39H1 | Validated | Human | gene with protein product |
| ATF2 | Validated | Human | gene with protein product |
| STK11 | Validated | Human | gene with protein product |
| CDH1 | Validated | Human, mice | gene with protein product |
| NEDD4L | Validated | Human | gene with protein product |
| FTMT | Validated | Human | gene with protein product |
| DECR1 | Validated | Human | gene with protein product |
| GLRX5 | Validated | Human, mice | gene with protein product |
| PANX2 | Validated | Human | gene with protein product |
| TFAP2A | Validated | Human | gene with protein product |
| CP | Validated | Human | gene with protein product |
| ARF6 | Validated | Human | gene with protein product |
| GDF15 | Validated | Human | gene with protein product |
| ABHD12 | Validated | Human | gene with protein product |
| TFAM | Validated | Human | gene with protein product |
| KDM3B | Validated | Human | gene with protein product |
| RNF113A | Validated | Human | gene with protein product |
| IDH2 | Validated | Human, mice | gene with protein product |
| PPARA | Validated | Human | gene with protein product |
| SIAH2 | Validated | Human | gene with protein product |
| NEDD4 | Validated | Human | gene with protein product |
| AR | Validated | Human | gene with protein product |
| MTF1 | Validated | Human | gene with protein product |
| COPZ1 | Validated | Human | gene with protein product |
| NUPR1 | Validated | Human | gene with protein product |
| USP35 | Validated | Human | gene with protein product |
| PDSS2 | Validated | Human | gene with protein product |
| BEX1 | Validated | Human | gene with protein product |
| FABP4 | Validated | Human | gene with protein product |
| TYRO3 | Validated | Human, mice | gene with protein product |
| SIRT6 | Validated | Human | gene with protein product |
| KIF20A | Validated | Human | gene with protein product |
| ECH1 | Validated | Human, mice | gene with protein product |
| ETV4 | Validated | Human | gene with protein product |
| VCP | Validated | Human | gene with protein product |
| RBMS1 | Validated | Human | gene with protein product |
| KDM4A | Validated | Human | gene with protein product |
| MGST1 | Validated | Human | gene with protein product |
| MPC1 | Validated | Human | gene with protein product |
| CAMKK2 | Validated | Human | gene with protein product |
| SOX2 | Validated | Human, mice | gene with protein product |
| SRSF9 | Validated | Human | gene with protein product |
| MEF2C | Validated | Human | gene with protein product |
| EZH2 | Validated | Human | gene with protein product |
| PEDS1 | Validated | Human | gene with protein product |
| CDC25A | Validated | Human | gene with protein product |
| LCN2 | Validated | Human | gene with protein product |
| TRIB2 | Validated | Human | gene with protein product |
| DHODH | Validated | Human | gene with protein product |
| PDK4 | Validated | Human | gene with protein product |
| PTPN18 | Validated | Human | gene with protein product |
| FTL | Validated | Human | gene with protein product |
| ABCC5 | Validated | Human | gene with protein product |
| CISD3 | Validated | Human | gene with protein product |
| GALNT14 | Validated | Human | gene with protein product |
| KLHDC3 | Validated | Human | gene with protein product |
| GSTM1 | Validated | Human | gene with protein product |
| RARRES2 | Validated | Human | gene with protein product |
| USP11 | Validated | Human | gene with protein product |
